# Application of machine learning model to predict lacunar cerebral infarction in elderly patients with femoral neck fracture before surgery

**DOI:** 10.1186/s12877-022-03631-1

**Published:** 2022-11-28

**Authors:** Cheng-bin Huang, Kai Tan, Zong-yi Wu, Lei Yang

**Affiliations:** 1 Department of Orthopaedic Surgery, The Second Affiliated Hospital and Yuying Childrens Hospital of Wenzhou Medical University, Wenzhou, 325000 China; 2 Key Laboratory of Orthopaedics of Zhejiang Province, Wenzhou, 325000 China

**Keywords:** Lacunar cerebral infarction, Femoral neck fracture, Machine learning, Old people, Prediction model

## Abstract

**Background:**

Femoral neck fracture and lacunar cerebral infarction (LCI) are the most common diseases in the elderly. When LCI patients undergo a series of traumas such as surgery, their postoperative recovery results are often poor. Moreover, few studies have explored the relationship between LCI and femoral neck fracture in the elderly. Therefore, this study will develop a ML (machine learning)-based model to predict LCI before surgery in elderly patients with a femoral neck fracture.

**Methods:**

Professional medical staff retrospectively collected the data of 161 patients with unilateral femoral neck fracture who underwent surgery in the Second Affiliated Hospital of Wenzhou Medical University database from January 1, 2015, to January 1, 2020. Patients were divided into two groups based on LCI (diagnosis based on cranial CT image): the LCI group and the non-LCI group. Preoperative clinical characteristics and preoperative laboratory data were collected for all patients. Features were selected by univariate and multivariate logistic regression analysis, with age, white blood cell (WBC), prealbumin, aspartate aminotransferase (AST), total protein, globulin, serum creatinine (Scr), blood urea nitrogen (Bun)/Scr, lactate dehydrogenase (LDH), serum sodium and fibrinogen as the features of the ML model. Five machine learning algorithms, Logistic regression (LR), Gradient Boosting Machine (GBM), Extreme Gradient Boosting (XGBoost), Random Forest (RF), and Decision tree (DT), were used in combination with preoperative clinical characteristics and laboratory data to establish a predictive model of LCI in patients with a femoral neck fracture. Furthermore, indices like the area under the receiver operating characteristic (AUROC), sensitivity, specificity, and accuracy were calculated to test the models’ performance.

**Results:**

The AUROC of 5 ML models ranged from 0.76 to 0.95. It turned out that the RF model demonstrated the highest performance in predicting LCI for femoral neck fracture patients before surgery, whose AUROC was 0.95, sensitivity 1.00, specificity 0.81, and accuracy 0.90 in validation sets. Furthermore, the top 4 high-ranking variables in the RF model were prealbumin, fibrinogen, globulin and Scr, in descending order of importance.

**Conclusion:**

In this study, 5 ML models were developed and validated for patients with femoral neck fracture to predict preoperative LCI. RF model provides an excellent predictive value with an AUROC of 0.95. Clinicians can better conduct multidisciplinary perioperative management for patients with femoral neck fractures through this model and accelerate the postoperative recovery of patients.

## Introduction

With the rapid development of medical technology in today’s world, the life expectancy of modern people continues to improve, and age-related diseases have become one of the main burdens of global medical expenditure. Femoral neck fracture and lacunar cerebral infarction (LCI) are the most common diseases in the elderly [1, 2]. Considering that the use of cannulated screw for reduction of femoral neck fracture requires a long time of bed immobilization and a high possibility of femoral head necrosis, clinicians generally recommend a hip replacement for elderly patients with femoral neck fracture [1, 3]. Hip replacement is a complex and challenging operation that requires strict perioperative management of patients, especially patients with cardiovascular and cerebrovascular conditions, to prevent serious complications such as pulmonary embolism [4].

Compared with non-LCI patients, LCI patients often experience additional problems after trauma, such as surgery [5, 6]. When patients with hip fractures are complicated by cerebral infarction, recovery of limb function is poor and postoperative mortality is higher [7, 8]. In addition, when fracture patients are complicated with cerebral infarction, multidisciplinary management often accelerates postoperative recovery and improves their quality of life [9, 10]. However, for elderly patients with a femoral neck fracture, head CT is not a routine preoperative examination to detect LCI in advance due to a series of economic factors. Therefore, there is an urgent need for a predictive model to predict whether elderly patients with femoral neck fractures are associated with LCI before surgery. Therefore, timely multidisciplinary management of patients with LCI can be carried out to promote the recovery of patients.

As a new algorithm model, the machine learning (ML) algorithm has been widely used in various fields, especially in the medical field [11]. The traditional ML model, Logistic regression (LR), Gradient boosting machine (GBM), Extreme gradient boosting (XGBoost), Random forest (RF) and Decision tree (DT) showed good predictive efficacy in different diseases [12, 13]. These prediction models provide better help for clinicians in making clinical decisions.

Therefore, this study will aim to develop a ML-based model to predict LCI before surgery in elderly patients with a femoral neck fracture. Clinicians will use ML model results to manage better perioperative management of elderly patients with a femoral neck fracture.

## Methods

### Study design

**Fig. 1:**
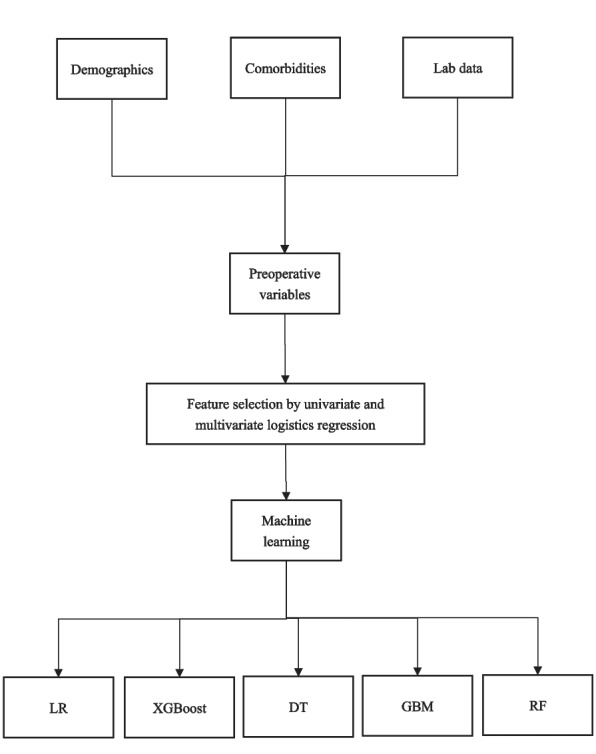
Flow chart showing analyses and model making process for the study. LR, Logistic regression; GBM, Gradient boosting machine; RF, Random forest; DT, Decision tree; XGBoost, Extreme gradient boosting

Variables including demographic characteristics, comorbidities, and laboratory data were collected from elderly patients with femoral neck fractures who underwent head CT. According to CT results, patients were divided into the LCI group and the non-LCI group. Univariate and multivariate logistic regression analyses were performed to select the preoperative variables of the two groups. The selected variables were placed into five machine learning models to predict LCI in patients with a femoral neck fracture. The detailed process is shown in Fig. 1.

### Setting

With the approval of the institutional review Committee, professional medical staff retrospectively collected and analyzed the data of patients with unilateral femoral neck fracture who underwent surgery in the database of the Second Affiliated Hospital of Wenzhou Medical University from January 1, 2015, to January 1, 2020.

### Participants

The inclusion criteria were:1) Unilateral femoral neck fracture, 2) No other fractures, 3) Age > 60 years, 4) LCI was diagnosed based on cranial CT images. The exclusion criteria were: 1) No CT scan of the head, 2) Previous history of brain disease, 3) With other fractures, 4) Pathological fracture, 5) Missing clinical characteristics or laboratory data.

### Variables

The study included 161 elderly patients (age > 60 years) with unilateral femoral neck fracture. Patients were divided into two groups based on lacunar cerebral infarct (diagnosis based on cranial CT image): the LCI (lacunar cerebral infarct) group and the non-LCI group. Preoperative clinical characteristics and preoperative laboratory data were collected for all patients. These preoperative variables include age, BMI (body mass index), injury mechanism, gender, types of admission, current drinking, current smoking, injured limb, coronary heart disease, hypertension, diabetes, fatty liver, kidney stone, gallbladder stone, pulmonary nodule, pneumonia, thrombus of lower limb, VTE (venous thrombus embolism), Barthel index [14], education level, prealbumin, total protein, albumin, globulin, A/G (albumin/globulin), AST (aspartate aminotransferase), ALT (alanine aminotransferase), ASL/ALT, ALP (alkaline phosphatase), GGT (gamma-glutamyltransferase), total bilirubin, direct bilirubin, indirect bilirubin, FBG (fasting blood glucose) cystatin-C, BUN (blood urea nitrogen), Scr (serum creatinine), BUN/Scr, CPK (creatine phosphate kinase), homocysteine, LDH (lactate dehydrogenase), blood uric acid, serum sodium, serum kalium, serum calcium, serum chlorine, WBC (white blood cell), neutrophils, lymphocyte, monocyte, eosinophilic granulocyte, basophilic granulocyte, RBC (red blood cell), hemoglobin, hematocrit, MCV (mean corpuscular volume), MCH (mean corpuscular hemoglobin), MCHC (mean corpusular hemoglobin concerntration), platelet count, plateletcrit, PDW (platelet distribution width), MPV (mean platelet volume), PT (prothrombin time), INR (international normalized ratio), APTT (activated partial thromboplastin time), TT (thrombin time), fibrinogen and d-dimer.

### Statistics

Data distribution was tested using the Shapiro-Wilk test. As appropriate, patient characteristics were described using median (interquartile range [IQR]) and mean ± standard deviation, frequency, and percentage. A nonparametric test (Mann-Whitney U test or Kruskal-Wallis test) was applied for data with non-normal distribution or heterogeneity of variances. Categorical variables were expressed as percentages and analyzed using the Pearson Chi-squared test. Univariate logistic regression analysis determined the independent risk factors for lacunar cerebral infarct. For the independent risk factors of LCI, the multivariable logistic regression included risk factors significantly associated with LCI in the univariate analysis (*P* < 0.1).

This study incorporated risk factors selected by univariate and multivariable logistics regression analysis into the machine learning model-like features. To make the ML model more accurate, we conducted zero-mean normalization of the data. Moreover, we randomly split our dataset into two groups: the training sets (70%) for ML model development and the validation sets (30%) for performance evaluation. Besides, we developed five types of ML algorithms to model our data: Logistic regression (LR), Gradient boosting machine (GBM), Extreme gradient boosting (XGBoost), Random forest (RF) and Decision tree (DT). In order to make the ML model more reliable and stable, we carried out a 10-fold cross-validation on the data set during training. Then the ML algorithms were further trained to predict the risk of LCI before surgery, and we evaluated the predictive ability of each ML classifier in validation sets where the area under the receiver operating characteristic (AUROC) value and the corresponding sensitivity, specificity, as well as overall accuracy of ML algorithms were all calculated. All statistics were calculated using SPSS software (version 26.0; SPSS Inc., Chicago, IL, USA) and Python 3.7.6 (Python Software Foundation, http://python.org).

## Results

### Baseline characteristics of the study population

After excluding a series of variables affecting the results of this study, such as the absence of head CT and the history of brain disease, 161 patients were included in this study, including 100 patients in the LCI group and 61 patients in the non-LCI group. There were statistically significant differences between the two groups in age, hypertension, Barthel index, VTE, prealbumin, total protein, albumin, globulin, BUN/Scr, LDH, cholinesterase, serum sodium, serum chlorine, WBC, Neutrophils, PT, TT, fibrinogen, and d-dimer (all *P* values < 0.05). Moreover, there were no statistically significant differences between the two groups in BMI, education level, gender, current drinking, current smoking, injury mechanism, injured limb, thrombus of the lower limb, types of admission, coronary heart disease, diabetes, pulmonary nodule, fatty liver, pneumonia, kidney stones, gallbladder stone, A/G, AST, ALT, AST/ALT, ALP, GGT, total bilirubin, direct bilirubin, indirect bilirubin, FBG, BUN, Scr, cystatin-C, CPK, homocysteine, blood uric acid, serum kalium, serum calcium, lymphocyte, monocyte, eosinophilic granulocyte, basophilic granulocyte, RBC, hemoglobin, hematocrit, MCV, MCH, MCHC, platelet count, plateletcrit, PDW, MPV, INR, and APTT (Details are shown in Table 1).

**Table 1:** Comparison of preoperative clinical characteristics and preoperative laboratory data between two groups

Variables	Non-LCI (61)	LCI(100)	*P* value
Age (years)	**81(72-85)**	**84(78-88)**	**0.009**
BMI	21.48(19.78-24.16)	22.15(20.72-23.88)	0.273
Gender			0.974
Female, n(%)	16(26.2)	26(26.0)	
Male, n(%)	45(73.8)	74(74.0)	
Injury mechanism			0.555
Traffic accident injuries, n(%)	6(9.8)	6(6.0)	
Fall injury, n(%)	55(90.2)	94(94.0)	
Types of admission			0.216
Outpatient, n(%)	39(63.9)	54(54.0)	
Emergency, n(%)	22(36.1)	46(46.0)	
Current drinking, n(%)	2(3.3)	12(12.0)	0.057
Current smoking, n(%)	2(3.3)	6(6.0)	0.691
Injured limb			0.120
Left, n(%)	40(65.6)	52(53.1)	
Right, n(%)	21(34.4)	46(46.9)	
Coronary heart disease, n(%)	8(13.1)	16(16.0)	0.618
Hypertension, n(%)	**28(45.9)**	**62(62.0)**	**0.046**
Diabetes, n(%)	16(26.2)	18(18.0)	0.215
Pulmonary nodule, n(%)	20(32.8)	40(40.0)	0.358
Fatty liver, n(%)	18(29.5)	24(24.0)	0.440
Kidney stone, n(%)	4(6.6)	16(16.0)	0.078
Gallbladder stone, n(%)	10(16.4)	18(18.0)	0.794
Pneumonia, n(%)	24(39.3)	52(52.0)	0.119
Thrombus of lower limb, n(%)	8(13.1)	14(14.0)	0.874
Barthel index	**55(45-60)**	**40(35-50)**	**< 0.001**
VTE			**0.035**
Low-risk, n(%)	2(3.3)	0(0)	
Medium risk, n(%)	0(0)	14(14.0)	
High risk, n(%)	59(96.7)	86(86.0)	
Education level			0.499
Illiteracy, n(%)	41(67.2)	62(62.0)	
Primary, n(%)	14(23.0)	26(26.0)	
Junior middle, n(%)	4(6.6)	8(8.0)	
High school or above, n(%)	2(3.3)	4(4.0)	
Prealbumin (mg/L)	**205(181-272)**	**164(134-206)**	**< 0.001**
Total protein (g/L)	**68.6(65.0-71.8)**	**64.8(59.8-68.5)**	**< 0.001**
Albumin (g/L)	**39.11 ± 3.34**	**37.54 ± 4.19**	**0.014**
Globulin (g/L)	**28.7(25.1-32.5)**	**26.5(23.9-28.9)**	**0.002**
A/G	1.33(1.22-1.61)	1.39(1.28-1.53)	0.361
AST (U/L)	24(19-26)	24(18-29)	0.324
ALT (U/L)	17(13-21)	17(11-22)	0.792
ASL/ALT	1.35 ± 0.40	1.44 ± 0.44	0.207
ALP (U/L)	82(63-101)	80(69-88)	0.520
GGT (U/L)	27(18-45)	29(13-37)	0.692
Total bilirubin (umol/L)	16.3(11.7-25.0)	16.0(10.8-21.1)	0.599
Direct Bilirubin (umol/L)	5.0(3.2-6.6)	4.8(3.1-6.5)	0.903
Indirect Bilirubin (umol/L)	12.7(7.4-16.2)	12.0(7.4-14.1)	0.297
FBG (mmol/L)	6.78(5.62-7.90)	6.45(5.61-7.34)	0.217
BUN (mmol/L)	7.2(5.7-8.9)	6.7(5.3-9.3)	0.505
Scr (umol/L)	62.9(48.3-77.7)	64.9(56.6-83.0)	0.078
BUN/Scr	**0.11(0.09-0.14)**	**0.10(0.08-0.12)**	**0.008**
Cystatin-C (mg/L)	1.25(1.09-1.38)	1.26(1.06-1.44)	0.616
CPK (U/L)	124(85-221)	138(90-220)	0.845
Homocysteine (umol/L)	14.2(9.1-15.7)	15.4(10.2-16.5)	0.174
LDH (U/L)	**251(225-291)**	**247(221-265)**	**0.041**
Cholinesterase (U/L)	**7592(6946-9107)**	**7592(5953-7642)**	**0.042**
Blood uric acid (umol/L)	329(304-376)	321(254-402)	0.541
Serum sodium (mmol/L)	**138.2(136.6-139.9)**	**139.3(137.8-141.4)**	**0.004**
Serum kalium (mmol/L)	3.87 ± 0.40	3.81 ± 0.46	0.387
Serum calcium (mmol/L)	2.20(2.14-2.27)	2.16(2.08-2.23)	0.068
Serum chlorine (mmol/L)	**103.04 ± 3.05**	**104.49 ± 3.20**	**0.005**
WBC (10^9/L)	**9.16 ± 2.95**	**8.16 ± 2.64**	**0.028**
Neutrophils (10^9/L)	**7.46 ± 2.67**	**6.59 ± 2.57**	**0.042**
Lymphocyte (10^9/L)	1.13(0.77-1.45)	0.97(0.67-1.41)	0.126
Monocyte (10^9/L)	0.41(0.34-0.49)	0.44(0.31-0.55)	0.284
Eosinophilic granulocyte (10^9/L)	0.07(0.02-0.15)	0.07(0.04-0.14)	0.907
Basophilic granulocyte (10^9/L)	0.010(0-0.013)	0.010(0-0.016)	0.952
RBC (10^12/L)	4.10(3.81-4.36)	3.96(3.53-4.33)	0.226
Hemoglobin (g/L)	126(116-132)	123(103-131)	0.328
Hematocrit	0.37(0.35-0.39)	0.37(0.35-0.39)	0.357
MCV (fl)	91.0(88.7-94.4)	91.4(89.3-94.1)	0.589
MCH (pg)	30.5(29.7-32.0)	30.9(29.7-32.1)	0.590
MCHC (g/L)	335.25 ± 9.03	335.90 ± 11.87	0.712
Platelet count (10^9/L)	196.21 ± 53.83	197.38 ± 57.06	0.898
Plateletcrit	0.18(0.16-0.23)	0.19(0.16-0.22)	0.564
PDW, n(%)	15.9(12.5-16.4)	15.8(13.1-16.2)	0.686
MPV (fl)	9.9(9.1-10.7)	9.8(9.3-10.5)	0.874
PT (seconds)	**13.8(12.9-14.8)**	**13.5(13.1-14.1)**	**0.020**
INR	1.12(0.98-1.18)	1.06(1.01-1.12)	0.056
APTT (seconds)	39.5(33.8-46.4)	39.4(35.8-42.6)	0.910
TT (seconds)	16.0(15.3-16.7)	15.5(14.7-16.7)	0.017
Fibrinogen (g/L)	**3.79(3.24-4.18)**	**4.66(3.73-5.69)**	**< 0.001**
D-Dimer (ug/ml)	6.99(2.61-13.61)	3.57(2.31-6.89)	0.001

Abbreviations: *BMI* Body mass index, *VTE* Venous thrombus embolism, *WBC* White blood cell, *RBC* Red blood cell, *MPV* Mean platelet volume, *AST* Aspartate aminotransferase, *ALT* Alanine aminotransferase, *A/G* Albumin/globulin, *CPK* Creatine phosphate kinase, *BUN* Blood urea nitrogen, *Scr* Serum creatinine, *MCHC* Mean corpusular hemoglobin concerntration, *ALP* Alkaline phosphatase, *GGT* Gamma-glutamyltransferase, *FBG* Fasting blood glucose, *LDH* Lactate dehydrogenase, *MCV* Mean corpuscular volume, *PDW* Platelet distribution width, *MCH* Mean corpuscular hemoglobin, *PT* Prothrombin time, *INR* International normalized ratio, *APTT* Activated partial thromboplastin time, *TT* Thrombin time

### Logistic regression analysis for independent risk factors of LCI in femoral neck fracture patients

The univariate logistics regressions analysis was applied to the baseline variables, laboratory tests, and comorbidities. Age, BMI, injury mechanism, gender, types of admission, current drinking, current smoking, injured limb, hypertension, diabetes, fatty liver, kidney stone, gallbladder stone, coronary heart disease, pulmonary nodule, pneumonia, thrombosis of the lower limb, Barthel index, VTE, education level, prealbumin, total protein, albumin, globulin, A/G, AST, ALT, ASL/ALT, ALP, GGT, total bilirubin, direct bilirubin, indirect bilirubin, FBG, BUN, Scr, BUN/Scr, cystatin-C, CPK, homocysteine, LDH, cholinesterase, blood uric acid, serum sodium, serum kalium, serum calcium, serum chlorine, WBC, neutrophils, lymphocyte, monocyte, eosinophilic granulocyte, basophilic granulocyte, RBC, hemoglobin, hematocrit, MCV, MCH, MCHC, platelet count, plateletcrit, PDW, MPV, PT, INR, APTT, TT, fibrinogen, and d-dimer were analyzed during the univariate analysis. The significant parameters (*p* < 0.1): age, current drinking hypertension, kidney stone, Barthel index, prealbumin, total protein, albumin, globulin, AST, FBG, Scr, BUN/Scr, LDH, cholinesterase, serum sodium, serum chlorine, WBC, neutrophils, PT, INR, TT, fibrinogen, and d-dimer were included in multiple logistic regression analysis (Table 2). The results showed that age, WBC, prealbumin, AST, Total protein, globulin, Scr, BUN/Scr, LDH, serum sodium and fibrinogen were independent predictors of LCI in femoral neck fracture patients (Table 3).

**Table 2:** Univariate logistics regressions analysis of risk factors to femoral neck fracture patients with lacunar cerebral infarct

Variables	OR	95%CI	*P*
Age (years)	1.058	1.017-1.099	0.005
Current drinking	4.023	0.869-18.631	0.075
Hypertension	1.923	1.008-3.667	0.047
Kidney stone	2.714	0.863-8.539	0.088
Barthel index	0.940	0.913-0.967	< 0.001
Prealbumin (mg/L)	0.982	0.975-0.989	< 0.001
Total protein (g/L)	0.905	0.857-0.955	< 0.001
Albumin (g/L)	0.899	0.824-0.981	0.017
Globulin (g/L)	0.909	0.849-0.972	0.006
AST (U/L)	1.032	0.999-1.067	0.056
FBG (mmol/L)	0.877	0.779-0.986	0.029
Scr (umol/L)	1.013	1.000-1.026	0.047
BUN/Scr	2E-06	2.24E-10-0.014	< 0.001
LDH (U/L)	0.994	0.988-1.001	0.087
Cholinesterase (U/L)	1.000	1.000-1.000	0.011
Serum sodium (umol/L)	1.220	1.068-1.394	0.003
Serum chlorine (umol/L)	1.158	1.042-1.286	0.007
WBC (10^9/L)	0.878	0.780-0.988	0.030
Neutrophils (10^9/L)	0.880	0.777-0.997	0.044
PT (seconds)	0.609	0.415-0.895	0.012
INR	0.024	0.001-0.775	0.035
TT (seconds)	0.819	0.665-1.010	0.062
Fibrinogen (g/L)	2.779	1.831-4.217	< 0.001
D-Dimer (ug/ml)	0.910	0.862-0.961	0.001

Abbreviations: *WBC* White blood cell, *AST* Aspartate aminotransferase, *BUN* Blood urea nitrogen, *Scr* Serum creatinine, *FBG* Fasting blood glucose, *LDH* Lactate dehydrogenase, *PT* Prothrombin time, *INR* International normalized ratio, *TT* Thrombin time

**Table 3:** Multivariate logistics regressions analysis of risk factors to femoral neck fracture patients with lacunar cerebral infarct

Variables	OR	95%CI	*P*
Age (years)	1.338	1.161-1.542	< 0.001
WBC (10^9/L)	0.425	0.262-0.691	0.001
Prealbumin (mg/L)	0.958	0.935-0.981	< 0.001
AST (U/L)	1.381	1.132-1.684	0.001
Total protein (g/L)	1.814	1.288-2.555	0.001
Globulin (g/L)	0.306	0.176-0.533	< 0.001
Scr (umol/L)	0.975	0.955-0.995	0.016
Bun/Scr	3.32E-29	1.19E-44-9.27E-14	< 0.001
LDH (U/L)	0.984	0.969-1.000	0.048
Serum sodium (umol/L)	1.694	1.148-2.498	0.008
Fibrinogen (g/L)	42.273	5.507-324.514	< 0.001

Abbreviations: *WBC* White blood cell, *AST* Aspartate aminotransferase, *BUN* Blood urea nitrogen, *Scr* Serum creatinine, *LDH* Lactate dehydrogenase

### Performance of machine learning algorithms

**Fig. 2:**
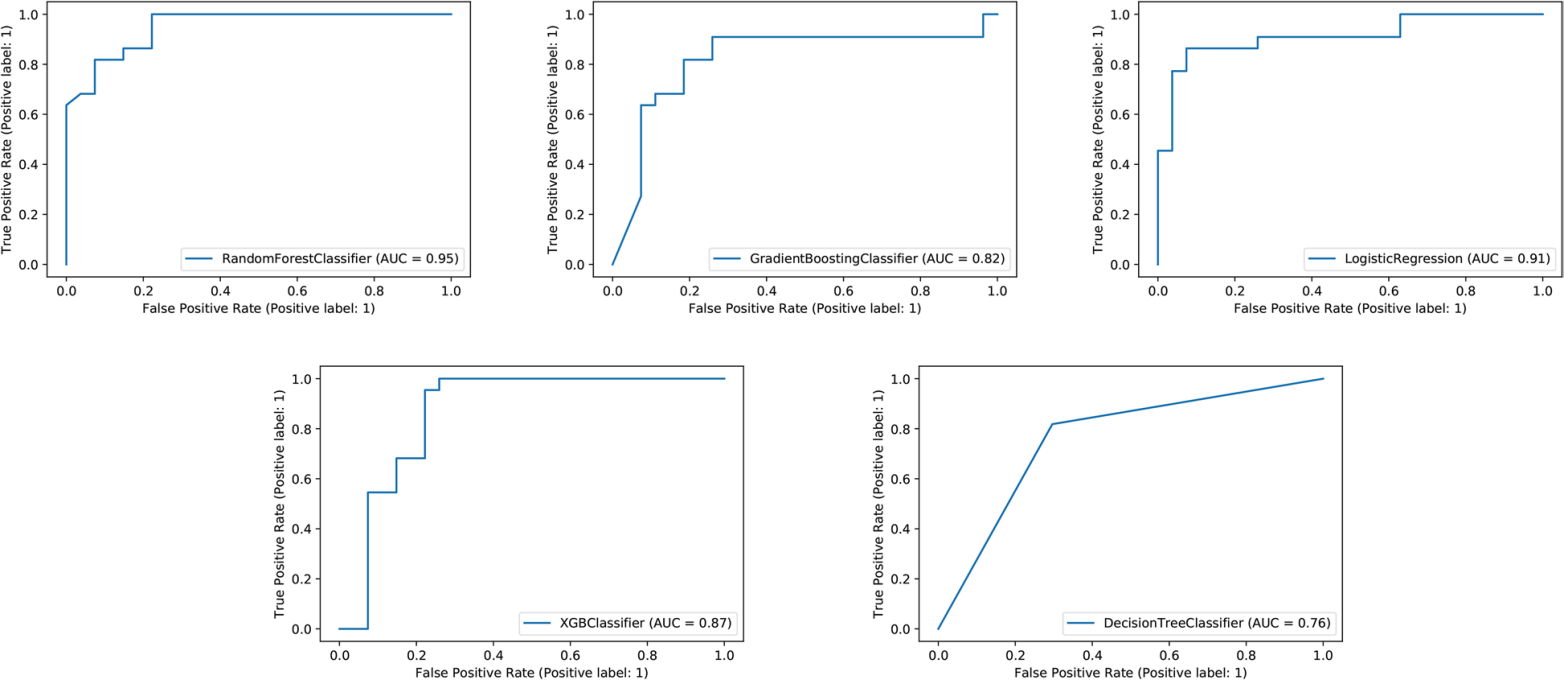
ROC curve analysis of machine learning algorithms for prediction of femoral neck fracture patients with LCI in the validation set. XGB, Extreme gradient boosting; ROC, receiver operating characteristic; AUC, area under the curve; LCI, lacunar cerebral infarction

Comparisons of the prediction performance among the 5 ML models in validation sets are detailed in Table 4 and Fig. 2. It turned out that the RF model demonstrated the highest performance in predicting LCI for femoral neck fracture patients before surgery, whose AUROC was 0.95, sensitivity 1.00, specificity 0.81, and accuracy 0.90 in validation sets.

**Table 4:** Predictive performance comparison of the five types of machine learning algorithms in the validation sets

Model	AUROC	Sensitivity	Specificity	Accuracy
LR	0.91	0.86	0.74	0.80
XGBoost	0.87	0.95	0.78	0.86
DT	0.76	0.82	0.70	0.76
GBM	0.82	0.82	0.78	0.80
RF	0.95	1.00	0.78	0.88

Abbreviations: *LR* Logistic regression, *GBM* Gradient boosting machine, *RF* Random forest, *DT* Decision tree, *XGBoost* Extreme gradient boosting

### Relative importance of variables in machine learning algorithms

**Fig. 3:**
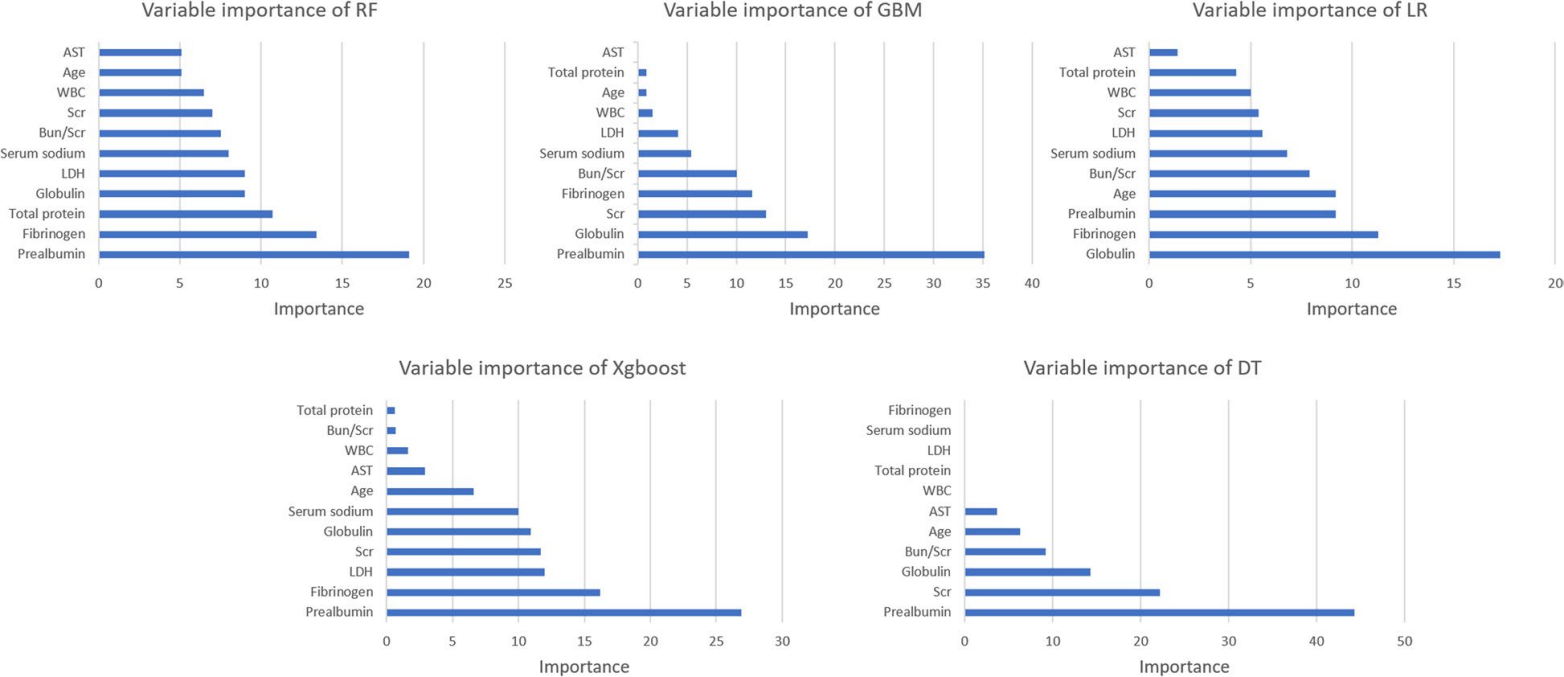
Relative importance ranking of each input variable for prediction of LCI in the machine learning algorithms. LR, Logistic regression; GBM, Gradient boosting machine; RF, Random forest; DT, Decision tree; XGBoost, Extreme gradient boosting; WBC, white blood cell; AST, aspartate aminotransferase; Scr, serum creatinine; Bun blood urea nitrogen; LDH, lactate dehydrogenase

The relative importance of variables in each LCI-predicting ML algorithm is shown in Fig. 3. There are general evidence trends: although the importance of variables in these ML algorithms varies slightly, factors including prealbumin, globulin, fibrinogen and Scr are more critical than other factors such as AST and WBC. The importance of high-ranking variables in the RF model is arranged in descending order: prealbumin, fibrinogen, globulin and Scr.

## Discussion

Previous studies [15–17] only discussed LCI-related risk factors such as renal function. However, these studies only identified risk factors closely associated with LCI and did not integrate these risk factors into a model to predict LCI. Compared with traditional models, machine learning has strong predictive power for classification problems and has been widely used in the medical field [11]. Therefore, to our knowledge, this study is the first to use a ML algorithm to predict whether an elderly patient with a femoral neck fracture has LCI before surgery.

In this study, our team used five popular machine learning models, LR, XGBoost, DT, GBM, and RT, to predict lacunar cerebral infarction in elderly patients with femoral neck fracture before surgery. To our satisfaction, these 5 ML models all showed excellent predictive ability, among which RF had the most robust predictive ability with an AUROC of 0.95. Therefore, clinicians can use the RF model to predict whether elderly patients with femoral neck complicated with LCI before surgery.

These 5 ML models confirmed that prealbumin, globulin, fibrinogen and Scr were important preoperative predictors of LCI in elderly patients with a femoral neck fracture. As a good reflection of the nutritional status of the human body, prealbumin has excellent predictive value for a variety of diseases, such as stroke [18, 19]. The results of this study are similar to those of previous studies [20], suggesting that prealbumin can be an effective indicator of LCI in elderly patients with a femoral neck fracture. Similarly, as an immune indicator, globulin has good predictive value in a range of diseases such as LCI [21, 22]. Zecca B et al. [23] found that globulin levels were strongly associated with ischemic stroke, which partially supports the findings of this study that globulin can predict LCI in elderly patients with a femoral neck fracture. Fibrinogen is closely related to thrombosis, and high fibrinogen levels are often regarded as biomarkers of vascular diseases such as myocardial infarction or stroke [24]. Some studies [25–27] have shown that high levels of fibrinogen can predict the occurrence and clinical prognosis of LCI, which further supports the results of this study, suggesting that fibrinogen can effectively predict the combination of LCI in elderly patients with a femoral neck fracture. Scr is one of the most commonly used indicators of kidney function. In addition, some studies have clarified the relationship between renal function and LCI [28–30]. Akoudad S et al. [31] demonstrated that renal function was closely related to the lesions of brain microvessels, especially the albumin-to-creatinine ratio. Similarly, Scr was a helpful predictor of LCI in elderly patients with a femoral neck fracture.

Compared with previous studies, this study has the following advantages. First, few studies have explored the risk factors associated with LCI and constructed predictive models. This study is the first to use an ML model to predict LCI in elderly patients with femoral neck fractures before surgery. Secondly, 5 ML models were used in this study to predict LCI, and the prediction effect was excellent. This supports the accuracy and reliability of the results of this study to a certain extent. Finally, the RF model had the most potent predictive power among the 5 ML models, with an AUROC of 0.95. Therefore, clinicians can use the RF model to predict whether elderly patients with femoral neck fracture have LCI before surgery, providing a basis for preoperative head CT examination and better perioperative management of patients.

However, this study has the following limitations. First, head CT is not a routine examination for elderly patients with femoral neck fracture in our institution, resulting in a relatively small number of subjects in this study. Secondly, this study is a retrospective cohort study, which will lead to a certain degree of bias. The ML model in this study consists of regression methods, so the model mainly shows which variable is the most predictive. In addition, the importance value of featured variables in the RF model in this study is relatively small, which may affect the effectiveness of RF to a certain extent. Therefore, a large prospective cohort study is urgently needed in the future to verify the results of this study. Finally, the ML model in this study was established based on patients in our institution, which may cause the lack of universality of the ML model. Therefore, data from external institutions are needed to verify the ML model of this study in the future.

## Conclusions

In this study, 5 ML models were developed and validated for patients with femoral neck fracture to predict preoperative LCI. RF model provides a good predictive value with an AUROC of 0.95. Clinicians can better conduct multidisciplinary perioperative management for patients with femoral neck fracture through this model and accelerate postoperative recovery of patients.

## Data Availability

The datasets analyzed in the study are available from the corresponding author on reasonable request.

## Abbreviations

BMI: body mass index
VTE: venous thrombus embolism
WBC: white blood cell
RBC: red blood cell
MPV: mean platelet volume
AST: aspartate aminotransferase
ALT: alanine aminotransferase
A/G: albumin/globulin
CPK: creatine phosphate kinase
BUN: blood urea nitrogen
Scr: serum creatinine
MCHC: mean corpusular hemoglobin concerntration
ALP: alkaline phosphatase
GGT: gamma-glutamyltransferase
FBG: fasting blood glucose
LDH: lactate dehydrogenase
MCV: mean corpuscular volume
PDW: platelet distribution width
MCH: mean corpuscular hemoglobin
PT: prothrombin time
INR: international normalized ratio
APTT: activated partial thromboplastin time
TT: thrombin time
LR: Logistic regression
GBM: Gradient boosting machine
RF: Random forest
DT: Decision tree
XGBoost: Extreme gradient boosting
LCI: lacunar cerebral infarction
ML: machine learning
